# Further Evidence of Possible Therapeutic Uses of *Sambucus nigra* L. Extracts by the Assessment of the In Vitro and In Vivo Anti-Inflammatory Properties of Its PLGA and PCL-Based Nanoformulations

**DOI:** 10.3390/pharmaceutics12121181

**Published:** 2020-12-04

**Authors:** Ana Henriques Mota, Noélia Duarte, Ana Teresa Serra, António Ferreira, Maria Rosário Bronze, Luísa Custódio, Maria Manuela Gaspar, Sandra Simões, Patrícia Rijo, Lia Ascensão, Pedro Faísca, Ana Silveira Viana, Rui Pinto, Pradeep Kumar, António José Almeida, Catarina Pinto Reis

**Affiliations:** 1 iMED, ULisboa, Research Institute for Medicines, Faculdade de Farmácia, Universidade de Lisboa, Av. Prof. Gama Pinto, 1649-003 Lisboa, Portugal; ana.luisa.mota@campus.ul.pt (A.H.M.); mduarte@ff.ulisboa.pt (N.D.); mrbronze@ff.ulisboa.pt (M.R.B.); mgaspar@ff.ulisboa.pt (M.M.G.); ssimoes@ff.ulisboa.pt (S.S.); p1609@ulusofona.pt (P.R.); rapinto@ff.ulisboa.pt (R.P.); aalmeida@ff.ulisboa.pt (A.J.A.); 2 IBET, Instituto de Biologia Experimental e Tecnológica, Av. da República, Estação Agronómica, Apartado 12, 2780-901 Oeiras, Portugal; tserra@ibet.pt (A.T.S.); antoniof@ibet.pt (A.F.); 3 Centre of Marine Sciences, Faculty of Sciences and Technology, University of Algarve, Ed. 7, Campus of Gambelas, 8005-139 Faro, Portugal; lcustodio@ualg.pt; 4 Centro de Estudos do Ambiente e do Mar (CESAM), Faculdade de Ciências, Universidade de Lisboa, Campo Grande, 1749-016 Lisboa, Portugal; lmpsousa@fc.ul.pt; 5 Faculdade de Medicina Veterinária—Universidade Lusófona de Humanidades e Tecnologias/DNAtech Laboratório Veterinário, Campo Grande 376, 1749-024 Lisboa, Portugal; pedrofaisca76@gmail.com; 6 Centro de Química Estrutural, Faculdade de Ciências, Universidade de Lisboa, Campo Grande, 1749-016 Lisboa, Portugal; apsemedo@fc.ul.pt; 7 Joaquim Chaves Saude. Dr. Joaquim Chaves, Laboratório de Análises Clínicas, 1495-068 Miraflores-Algés, Portugal; 8 Department of Pharmacy and Pharmacology, School of Therapeutic Sciences, Faculty of Health Sciences, University of the Witwatersrand, Johannesburg, 7 York Road, Parktown 2193, South Africa; Pradeep.Kumar@wits.ac.za; 9 IBEB, Biophysics and Biomedical Engineering, Faculdade de Ciências, Universidade de Lisboa, 1749-016 Lisboa, Portugal

**Keywords:** *Sambucus nigra* L., nanoparticles, anti-inflammatory activity, collagenase inhibition, safety assessment

## Abstract

*Sambucus nigra* L. is widely used in traditional medicine with different applications. However, confirmative studies are strongly required. This study aimed to assess the biological activities of the *S. nigra* flower’s extract encapsulated into two different types of nanoparticles for optimizing its properties and producing further evidence of its potential therapeutic uses. Different nanoparticles (poly(lactide-co-glycolide, PLGA) and poly-Ɛ-caprolactone (PCL), both with oleic acid, were prepared by emulsification/solvent diffusion and solvent-displacement methods, respectively. Oleic acid was used as a capping agent. After the nanoparticles’ preparation, they were characterized and the biological activities were studied in terms of collagenase, in vitro and in vivo anti-inflammatory, and in vitro cell viability. Rutin and naringenin were found to be the major phenolic compounds in the studied extract. The encapsulation efficiency was higher than 76% and revealed to have an impact on the release of the extract, mainly for the PLGA. Moreover, biochemical and histopathological analyses confirmed that the extract-loaded PLGA-based nanoparticles displayed the highest anti-inflammatory activity. In addition to supporting the previously reported evidence of potential therapeutic uses of *S. nigra*, these results could draw the pharmaceutical industry’s interest to the novelty of the nanoproducts.

## 1. Introduction

In recent years, plants have become a great source of bioactive compounds, mainly due to their biological activities. Their applicability is vast, and is suitable in many areas of the pharmaceutical and cosmetic industries [1]. The literature suggests that natural products play an important role in photo-aging prevention (e.g., olive oil), topical application of antioxidants (e.g., vitamins C and E) in the protection of skin against UV-mediated damage [1,2], among many others.

*Sambucus nigra* L. is one of medicinal plants that has been used since ancient times [3,4]. In our previous study, some important biological activities have been found in twenty-two extracts of *S. nigra* flowers (elderflowers) and berries (elderberries) and one of them suggested potential anti-inflammatory activity [5,6]. Thus, the present study reports for the first time the combination of a methanolic extract of *S. nigra* flowers with polymeric nanoparticles (NPs) for potential topical use. The strategy of using NPs as a carrier for extracts has been successfully developed in many fields, because NPs present several advantages, such as protection from moisture, light, heat and/or oxidation, as well as modifying the release of incorporated or loaded compounds [7]. In the current study, two polymers were used to prepare NPs: poly(lactide-co-glycolide) (PLGA) and poly-Ɛ-caprolactone (PCL). To the best of our knowledge, and specifically for *S. nigra,* a small number of studies combining *S. nigra* and NPs are described in the literature. In one study, an acetone: water extract of the black fruit elderberries is reported to present a potential interest to the treatment of psoriasis lesions [8] and in other study the encapsulation of the fruit’s extract in gold NPs is described for diabetes treatment [9]. The encapsulation of a dried aqueous extract of elderberries in liposomes is also mentioned in the literature [10]. The encapsulation efficiency was around 25% but no therapeutic applications of this combination were tested. In all three cited examples, none described the use of extracts obtained from elderflowers with NPs.

Thus, taking advantage of the biological properties of the methanolic extract of elderflowers [5,6] and of the combination with the carriers’ nanoparticulate nature, a new nanoformulation is herein proposed as an alternative to the classical anti-inflammatory products in the market. Extract-loaded PLGA NPs and extract-loaded PCL NPs, both with oleic acid, were prepared and characterized in terms of mean size, size distribution, surface charge, morphology, encapsulation efficiency of the extract and release. Total polyphenol content (TPC), antioxidant activity (AA), collagenase (Coll) inhibition, safety and in vitro and in vivo anti-inflammatory (AIA) were also investigated.

## 2. Materials and Methods

### 2.1. Materials

#### 2.1.1. Plant Materials

*Sambucus nigra* L. flowers (elderflowers) were supplied by Régiefrutas—Cooperativa Agrícola de Interesse Público Távora-Varosa, CIPRL, collected from commercial crops at Tarouca, Beira Alta, Portugal (lat. 40°59′06″ N; long. 7°37′03″ W; 695 m alt.) in May 2019.

#### 2.1.2. Chemicals

Poly(lactide-co-glycolide) (Purasorb^®^ PLDG 5002-PLGA Ratio L/G % 50:50; molecular weight 45.000–75.000 Da) was supplied by Purac Biomaterials (Gorinchen, The Netherlands) and oleic acid by FlukaChemika (Buchs, Swiss). Poly-Ɛ-caprolactone’s molecular weight is 14.000 g/mol (PCL), Pluronic^®^ F127 (PF127), collagenase (Coll) from *Clostridium histolyticum* type IA, 2,2-diphenyl-1-picrylhydrazyl (DPPH), Dulbecco’s Modified Eagle’s Medium—high glucose (DMEM), ascorbic acid, quercetin, Thiazolyl Blue Tetrazolium Bromide (MTT), (±)-6-Hydroxy-2,5,7,8-tetramethyl-chromane-2-carboxylic acid (Trolox), 2,2′-azobis(2-amidinopropane) dihydrochloride (AAPH), fluorescein sodium salt, hydrogen peroxide solution (H_2_O_2_), and Iron(III) chloride (FeCl_3_) were purchased from Sigma Aldrich (Steinheim, Germany). Roswell Park Memorial Institute (RPMI) 1640 medium, Iscove’s Modified Dulbecco’s Medium (IMDM—GlutaMAX™), and PenStrep were acquired from Gibco (Carlsbad, CA, USA). Fetal bovine serum (FBS) was supplied by Biowest (Riverside, CA, USA). PrestoBlue^®^ Cell Viability Reagent was obtained from Molecular Probes, Invitrogen (Waltham, MA USA). L-glutamine was supplied by Lonza (Leuven, Belgium). Rutin was acquired from Extrasynthese (Genay, France). Ketamine (Imalgene^®^ 1000) was purchased from Merial (Lyon, France), medetomidine (Medetor^®^, 1 mg/mL) from CP-Pharma (Handelsges, Germany). Commercial diclofenac sodium gel (23.2 mg/g) was acquired in a local pharmacy. Carbopol 940^®^ was purchased from Fagron (Barcelona, Spain). Epigallocatechingallate (EGCG) and *N*-[3-furyl-acryloyl]-Leu-Gly-Pro-Ala (FALGPA) were purchased from Panreac (Barcelona, Spain). Tricine buffer was purchased from VWR (Leuven, Belgium). Folin reagent and uranyl acetate were supplied by Merck (Darmstadt, Germany). High performance liquid chromatography (HPLC) grade acetonitrile and formic acid were obtained from Chem-Lab NV (Zedelgem, Belgium). Milli-Q water (18.2 MΩ cm^−1^ resistivity) was obtained from a Millipore-Direct Q3 UV system (Millipore^®^, Burlington, MA, USA). All other chemicals were of analytical grade.

#### 2.1.3. Biological or Cell Lines

RAW264.7 cells were obtained from Center for Neurosciences and Cell Biology, University of Coimbra, Coimbra, Portugal. HaCaT cell line was supplied by Cell-Line-Service cat: 300493, Eppelheim, Germany. Human fibroblast (HFF) cells were purchased from American Type Culture Collection (ATCC, Manassas, VA, USA).

#### 2.1.4. Animals

Adult female albino Wistar rats weighing around 200 g were supplied from the Instituto de Higiene e Medicina Tropical (Lisboa, Portugal). They were housed in polypropylene cages at room temperature (RT, 20–24 °C), with relative humidity (55 ± 5%), a 12 h light/dark cycle, and received a standard diet and water *ad libitum*. All animal experiments were conducted in line with the recommendations of the animal welfare board (ORBEA) of the Faculty of Pharmacy, Universidade de Lisboa, as well as approved by the competent national authority Direção-Geral de Alimentação e Veterinária (DGAV) (Protocol EXPL/DTP-FTO/0308), and in accordance with the EU Directive (2010/63/EU), the Portuguese laws (DL 113/2013, 2880/2015, 260/2016 and 1/2019), and all relevant legislation.

### 2.2. Methods

#### 2.2.1. Extraction

The extract was obtained from fresh elderflowers. MeOH was used as a solvent by the ultrasonication method (Sonorex Super RK 510 H; Bandelin, Berlin, Germany) which was performed according to Rijo et al. (2014) during 1 h and repeated three times, until the complete extraction [6,11]. Then, the extract was filtered and the MeOH was removed by rotary evaporation (Rotary evaporator from Heidolph type VV2000, Apeldoorn, Netherlands).

#### 2.2.2. Quantification of Major Compounds

HPLC-ESI-MS/MS assays were carried out on a Waters^®^ Alliance 2695 HPLC system (Waters^®^, Dublin, Ireland) coupled to a 2996 Photodiode Array Detector and a Micromass^®^ Quattro Micro triple quadrupole (TQ) (Waters, Dublin, Ireland). Chromatographic analyses were performed on a LiChrospher^®^ 100 RP-18 (250 × 4 mm, 5 μm) column at 35 °C. Mobile phase consisted of a mixture of formic acid (0.5% *v/v* in ultrapure water, eluent A) and 0.5% formic acid in acetonitrile (eluent B) at a flow rate of 0.3 mL/min. The following elution program was used: 20% B (0–5 min), changed linearly to 90% B (5–15 min), decreasing to 20% B in 1 min, and finally maintaining the initial conditions for 20 min as a re-equilibration step. The total running time was 40 min and the injection volume was 10 µL.

As reference, rutin and naringenin were previously identified as the major flavonoids of the extract [6]. MS/MS conditions were optimized for the identification and quantification of these compounds. The electrospray ion source (ESI) was set to operate at 120 °C in negative (rutin) or positive (naringenin) modes, using a capillary voltage of 2.5 kV, cone voltage of 30 V and collision energies of 18, 32, and 34 eV. High-purity nitrogen was used for drying and as a nebulizing gas. Ultra-high purity argon was used as a collision gas. In order to achieve a higher selectivity and sensitivity, analyses were performed in multiple reaction monitoring mode (MRM) to select the two product ions with the highest signal as the monitored transitions for quantification (MRM1, 609.00 > 300.00 for rutin and 237.00 > 153.00 for naringenin) and confirmation (MRM2, 609.00 > 271.00 for rutin and 237.00 > 147.00 for naringenin ) purposes. The Mass Lynx Version 4.1 software (Waters) was used for instrument control, data acquisition and data processing.

#### 2.2.3. Residual Quantity of Methanol

The residual methanol in the extract was analyzed by solid-phase microextraction, followed by gas chromatography-mass spectrometry method (SPME-GC-MS) carrying out a gas chromatography (GC) associated with MS with autosampler. The GC–MS equipment consisted of a QP 2010 Plus, Shimadzu (Kyoto, Japan), equipped with TeknoKroma Sapiens Wax-MS column 60 m × 0.25 mm i.d., film thickness 0.25 μm and an AOC-5000 Shimadzu autosampler (experimental conditions: carrier gas helium; column flow 4 mL/min; split injection with a ratio split of 1:50 and a temperature gradient; ionization energy, 70 eV; scan range, 29–300 m/z; detector and ionization temperatures, 250 °C). The following gradient of temperature was used for the separation of sample components: the column temperature was initially maintained at 40 °C for 1 min, then increased at a rate of 60 °C per minute to 150 °C and programmed to rise up to 260 °C at a rate of 45 °C per minute, and then maintained at that temperature for 5 min. Approximately 1.9892 g of the sample was measured to a capsulated vial (10 mL) and concentrated using solid phase microextration (SPME). A fiber of Divinylbenzene/Carboxen/Polydimethylsiloxane (DVB/CAR/PDMS) from Supelco (Bellefonte, PA, USA), 23 Ga, 50/30 μm, gray fiber assembly was used. The sample was heated for 40 min at 40 °C and injected at 250 °C. The time of desorption was 5 min. The identification of the components was assigned by comparing their retention indexes, and GC–MS spectra from the libraries (NIST 21, 27, 107, 147, and Wiley 229). The standard solutions of methanol in water were prepared at different conditions 1100, 2100, 3015, 4195, 5050 and 6060 ppm, and the calibration curve was performed with these six working standard solutions. Furthermore, the sample and the reference were analyzed by direct method using a reference of MeOH with a known concentration (3025 ppm). The calibration curve presented a y = 4E + 06× + 6E +06, with an R^2^ of 0.9955. Data acquisition was done using Shimadzu software, GC-MS solution, version 4.50 SP1.

#### 2.2.4. Preparation of Extract-Loaded Nanoparticles

To encapsulate the extract, two different NPs containing oleic acid as a capping agent to control NPs growth and aggregation, were developed, according to previous studies [12,13].

PLGA NPs were prepared by emulsification/solvent diffusion [5,13,14]. In brief, 50 mg PLGA was dissolved in a mixture acetone: ethanol (8:2, *v*/*v*) was added into an aqueous phase consisting of 0.1% PF127 and oleic acid (11 µL) under magnetic stirring (400 rpm), during 2 h [5,15]. NPs were instantaneously formed. The solvent-displacement method was used for PCL NPs [12]. In brief, an organic phase was prepared by mixing PCL and acetone. An aliquot of stearic acid (SA) in ethanol was added to the organic phase. This solution was immediately added to 10 mL aqueous solution of a PF127. Finally, 80 µL of oleic acid were added [12]. In both cases, the resulting NPs were centrifuged at 10,460× *g* (Sigma Laborzentrifugen, Osterode am Harz, Germany) for 15 and 20 min for PLGA and PCL NPs, respectively, at room temperature (RT), and subsequently resuspended in MilliQ water.

Extract-loaded NPs were prepared using the same methods, with an amount of extract corresponding to polymer: extract of 1:1 (*w/w*) and stored at refrigeration conditions (RC) (4 ± 2 °C).

#### 2.2.5. Physical Characterization of Nanoparticles

The mean size, the polydispersity index (PdI), the zeta potential and the pH of empty and extract-loaded NPs were evaluated over 12 months [12]. The NPs’ zeta potential was measured in a NaCl (0.1 M) solution for 12 months, and the mean size and PdI were measured in MilliQ water. The size, PdI and zeta potential were measured in diluted samples (1:10, *v*/*v*) by dynamic light scattering (DLS), using a Malvern Zetasizer Nano-S and Nano-Z (Malvern Instruments, Worcestershire, UK). Results were expressed as the mean of the measurements in triplicate. For this assay, empty and extract-loaded NPs were stored at three different temperatures, which is in line with ICH Q1A’s guideline (4 ± 2 °C—RC, 25 ± 5 °CRT and 40 ± 2 °C—accelerated conditions (AC)). Results are expressed as the mean ± S.D. (*n* = 3). The obtained suspensions of NPs were also characterized in terms of pH (827 pH Lab, Metrohm, Switzerland).

#### 2.2.6. Morphological Characterization

Particle’s morphology was characterized by scanning and transmission electron microscopy (SEM and TEM, respectively). For SEM, aliquots (20 µL) of the aqueous suspensions containing empty and extract-loaded PLGA and PCL NPs were uniformly dispersed over round glass coverslips, which were previously coated with poly-L-lysine and attached to the microscope stubs. Then, the samples were left in a desiccator for drying in dust free conditions, and subsequently coated with a thin layer of gold. Observations were carried out on a JEOL 5200LV scanning electron microscope (JEOL Ltd., Tokyo, Japan) at an accelerating voltage of 20 kV and images were recorded digitally.

For the TEM observations, aliquots (10 µL) of the empty and extract-loaded NPs suspensions were placed on Formvar/carbon coated grids for some minutes to allow the NPs’ adsorption. After removing the excess of the samples with filter paper, the material was negatively stained with 1% of uranyl acetate and left to air dry. Observations were made on a JEOL 1200EX transmission electron microscope (JEOL Ltd., Tokyo, Japan) operating at 80 kV. Images were recorded digitally.

The image software NanoScope V8 and Adobe^®^ Photoshop^®^ CS6 version 13.0 × 64 (Adobe Systems Pty. Ltd., Sydney, Australia) were used to prepare SEM and TEM figures.

For the atomic force microscopy (AFM) analysis, the samples were prepared by centrifugation using 7378× *g* (Sigma Laborzentrifugen, Osterode am Harz, Germany) for 5 and 10 min, for empty and extract-loaded NPs, respectively, followed by resuspension in water (for half of the previous volume) [13]. AFM uses diluted samples (1:2, *v*/*v*) without any pre-treatment. A drop of ≈40 μL of the sample was placed on a freshly cleaved mica surface to allow the absortion for around 30 min [13]. Afterwards, the samples were dried under a stream of N_2_ and analyzed in intermittent mode (Nanoscope V, Multimode 8 HR Microscope, produced by Bruker, Billerica, MA, USA) [13]. The images were recorded at ambient conditions (≈21 °C), using etched silicon tips with a resonance frequency of around 320 kHz (NCHV, Bruker, Billerica, MA, USA), at a scan rate of approximately 1.3 Hz [13]. All images were recorded digitally.

#### 2.2.7. Encapsulation Efficiency

The encapsulation efficiency (*EE*) was determined by using the same method referred in Section 2.2.2 and by analyzing the major compound present in supernatant. According to our previous assays, rutin was the major compound [6]. *EE* value was determined by Equation (1):

(1) $$EE=\;\left(\frac{Total\;amount_{bioactive\;compound}-Amount\;free_{bioactive\;compound}}{Total\;amount_{bioactive\;compound}}\right)\times 100$$

#### 2.2.8. Rutin and Nanoparticles’ Complexes

The reactional profile was assessed in silico via molecular mechanics simulations. The ensuing energetic-geometric stabilization provided an insight into the chemical interactions between the extract (major compound, rutin) and the NPs’ material (PLGA or PCL). An energetic and geometrical stabilization of the rutin-polymer molecular complexes was conducted using atomistic simulations (HyperChemLite Molecular Modelling Software, Hypercube Inc., Gainesville, FL, USA). The structures of PLGA, PCL and rutin were generated as natural bond angles. The individual molecules and the biomolecular complexes (PLGA-Rutin and PCL-Rutin) were energy minimized and optimized by employing the MM3 Force Field algorithm, which was then supported by a Polak–Ribiere Conjugate Gradient method until a Root Mean Square (RMS) gradient of 0.001 kcal/mol was achieved [16,17].

#### 2.2.9. In Vitro Release of the Extract from PLGA and PCL Nanoparticles

Extract-loaded NPs were previously freeze-dried for 24 h at −50 °C (Freezone 2.5 L, Freeze-dryer Labconco, Kansas City, MO, USA). Then, they were placed into amber glass bottles containing a phosphate buffer solution (PBS) (USP41) at pH 5.5 (10 mL) to simulate human skin pH, and stirred (200 rpm) at 32 °C in a multiplate stirring plate. The sink’s conditions were maintained during the whole assay. At pre-established time intervals (5 and 30 min, 1, 2, 4, 8 and 24 h), aliquots of the release medium (3 mL) were collected and immediately replaced with fresh buffer. The aliquots were centrifuged at 10,460× *g* (Sigma Laborzentrifugen, Osterode am Harz, Germany) for 15 and 20 min for PLGA and PCL NPs, respectively, and subsequently resuspended in MilliQ water. The extract concentration at each time point was determined through HPLC-ESI-MS/MS using rutin as reference, as described in Section 2.2.2. The same assay was performed in PBS (USP41) at pH 7.4 and 37 °C, as previously described. A standard calibration curve was performed with the rutin solution in PBS buffer pH 5.5 or 7.4 (USP41), depending on the assay performed. The calibration curve presented a range of concentrations of 10–1000 ppb from a stock solution of 1 mg/mL.

#### 2.2.10. Total Polyphenol Content

The total polyphenol content (TPC) was determined for the extract and extract-loaded NPs. It was estimated by using a modification of the Folin-Ciocalteu method [2]. In brief, a 100 μL aliquot of sample was added to 1 mL of Na_2_CO_3_ (15%, *w*/*v*), and 2 mL of water. Then, 200 μL of Folin-Ciocalteu reagent was added to each sample. The extracts were incubated for 1 h at RT and protected from light. Abs was measured against a blank in UV-Visible (UV-vis) spectrophotometer (Hitachi, Tokyo, Japan) at 765 nm, using a calibration curve of gallic acid in a concentration range of 6 to 500 mg/L. The results were expressed in milligrams of gallic acid equivalents per mL of sample (GAE; mean ± S.D., *n* = 3) [18].

#### 2.2.11. Antioxidant Activity

Different methods to evaluate the antioxidant activity (AA) of extract-loaded NPs were used: 2,2-diphenyl-1-picrylhydrazyl (DPPH), oxygen radical absorbance capacity (ORAC) and hydroxyl radical scavenging capacity (HOSC).

- 2,2-Diphenyl-1-picrylhydrazyl Assay (DPPH)

For this purpose, 10 mg of extract was dissolved in 1 mL of MeOH. Aliquots of 10 μL of each solution (free extract, positive controls and extract-loaded NPs) were diluted in 990 μL of a DPPH solution in ethanol 70% (*v*/*v*). The reaction mixture was incubated at RT for 30 min in the dark. After incubation, the absorbance (*Abs*) was measured at 517 nm (Thermo Scientific Evolution 300 UV-vis, Loughborough, UK). The positive controls consisted of a 10 mg/mL of quercetin solution in ethanol and 10 mg/mL of ascorbic acid solution in water. An absorbance control (*Abs_control_*) containing 10 μL of ethanol, and 990 μL of DPPH was prepared. The rutin concentration was 0.75 mg/mL in ethanol. This study was performed in triplicate (*n* = 3) and the free radical scavenging effect was then calculated according to Equation (2):

(2) $$Scavenging\;activity\;\left(\%\right)=\frac{Abs_{control}-\;Abs_{sample}}{Abs_{control}}\times 100$$

- Oxygen Radical Absorbance Capacity Assay (ORAC)

The ORAC’s method evaluates the samples’ antioxidant capacity to peroxyl radicals, based on previous works [19], but modified in order to use fluorescein (FL)× 800 microplate fluorescence reader (Bio-Tek Instruments, Winooski, VT, USA) [20]. In this method, the measurements of AA were made against peroxyl radical induced by 2,2′-azobis(2-amidinopropane) dihydrochloride (AAPH) at 37 °C [21]. This assay consists of a measure of the samples’ ability (antioxidant species) through the inhibition of the FL’s (3 × 10^−4^ mM) oxidation catalysed by AAPH [20]. The consumption of FL associated to its incubation with AAPH was estimated by fluorescence (F) measurements at 538 nm on excitation at 480 nm (Genios Spectrofluorimeter, Helvetic Tecan Group Ltd., Männedorf, Zürich, Switzerland) for a period of 40 min [22]. The values of instantaneous fluorescence/initial fluorescence (F/F_0_) were plotted in function of the time. The integration of area under curve (AUC) was performed for each time that F/F0 reached a value of 0.2 or 0.4 [23]. A reaction mixture containing AAPH (41.4 mg/mL), FL (4 × 10^−3^ mM) and the tested samples (free extract, extract-loaded PLGA NPs and extract-loaded PCL NPs) was incubated in phosphate buffer solution (PBS, 75 mM, pH 7.4) (USP41) at 37 °C [23]. PBS was used as a blank and 5, 10, 20, 30, and 40 µM Trolox solutions were used as control standards. All the samples, including the blank and the controls, were analyzed in triplicate. The final ORAC’s values were calculated by using a regression equation between the Trolox concentration and the net area under the FL decay curve [22]—Equation (3):

(3) $$ORAC-FL=\frac{AUC_{sample}-\;AUC_{0}}{AUC_{Trolox}-\;AUC_{0}}\times f\left[Trolox\right]$$

where *AUC_Sample_* is the *AUC* in the presence of the tested sample, integrated between time zero, which corresponds to 60% or 80% of the probe consumption; *AUC*_0_ is the *AUC* for the control (target molecule plus AAPH solution); *AUC_Trolox_* is the *AUC* for Trolox; f is the dilution factor, equal to the ratio between the total volume of the working solution (target molecule plus AAPH and the sample aliquot) and the added sample volume; [Trolox] is the Trolox molar concentration. Data were expressed as micromoles of Trolox equivalents antioxidant capacity (TEAC) per g of sample (extract, rutin or per NPs) [18,22]. The degraded extract-loaded NPs were also analyzed. The experiments were performed in triplicate [22].

- Hydroxyl Radical Scavenging Capacity Assay (HOSC)

The hydroxyl radical scavenging capacity (HOSC) was based on a previously reported method [24], which was slighty modified in order to adapt it for the FL× 800 microplate fluorescence reader (Bio-Tek Instruments, Winooski, VT, USA) [20]. A reaction mixture containing FeCl_3_ (3.42 mM), fluorescein (9.96 × 10^−8^ mM), H_2_O_2_ (0.20 M) and the tested samples (free extract, extract-loaded PLGA NPs and extract-loaded PCL NPs in the same concentrations as in the previous assay), was incubated in sodium phosphate buffer (SPB, 75 mM, pH 7.4, USP41) and mixture acetone: water (1:1) at 37 °C [23]. Mixture acetone: water was used as a blank and 5, 10, 15, 20, and 30 µM Trolox solutions were used as control standards. All the samples, including the blank and the controls, were analyzed in triplicate [22]. This assay evaluates the samples using fluorescein (9.96 × 10^−8^ mM) as a probe and a classic Fenton reaction with FeCl_3_ and H_2_O_2_ as a hydroxyl radicals [20]. To obtain HOSC-fluorescence (*HOSC-FL*) values, according to Equation (4):

(4) $$HOSC-FL=\frac{AUC_{sample}-\;AUC_{0}}{AUC_{Trolox}-\;AUC_{0}}\times f\left[Trolox\right]$$

As in Equation (3), here the *AUC_Sample_* is the *AUC* in the presence of the tested sample, integrated between time zero, which corresponds to 60% or 80% of the probe consumption; *AUC_0_* is the *AUC* for the control (target molecule more FeCl_3_ and H_2_O_2_); *AUC_Trolox_* is the *AUC* for Trolox; f is the dilution factor, equal to the ratio between the total volume of the working solution (target molecule more FeCl_3_ and H_2_O_2_ and the sample aliquot) and the added sample volume; [Trolox] is the Trolox molar concentration. Data were expressed as micromoles of Trolox equivalents antioxidant capacity (TEAC) per g of sample (extract, rutin or NPs) [18]. The degraded extract-loaded NPs were also analyzed. The experiments were performed in triplicate [22].

#### 2.2.12. In Vitro Collagenase Inhibition Activity

The collagenase (Coll) inhibition assay was performed in 50 mM tricine buffer (pH 7.5 with 400 mM NaCl, and 10 mM CaCl_2_). Coll from *Clostridium histolyticum* (EC.3.4.23.3) was dissolved in buffer at an initial concentration of 0.8 Units/mL, according to the supplier’s activity data. The synthetic substrate FALGPA was dissolved into 2 mM in tricine buffer and placed in a 96-well plate. Negative controls were tested at same concentration (1 mg of extract/mL and 1 mg of extract/mL of NPs suspension, according to EE). Abs was immediately measured at 405 nm after adding substrate, and then it was continuously measured for 10 min using a microplate reader (Thermo Scientific Multiskan FC, Shanghai, China). The EGCG was used as a positive control at a concentration of 250 μM. The Coll inhibition (%) was determined using Equations (5) and (6):

(5) $$Velocity\;reaction\;of\;control\;or\;inhibitor=\;\frac{Corrected\;Abs}{time\;\left(min\right)}$$

(6) $$Collagenase\;inhibition\;activity\;\left(\%\right)=100-\;\left(\frac{100\;\times Velocity\;reaction\;of\;inhibitor}{Velocity\;reaction\;of\;control}\right)$$

For the Coll activity, the *Abs* decrease was calculated by using Equation (5) for the velocity reaction of negative control (Δ*Abs_405nm_/min*). Then, to determine the Coll’s inhibitions activity, Equation (6) was used.

#### 2.2.13. In Vitro Safety Assessment for Potential Topical Use

- Cell Viability of Human Keratinocyte Cells

The potential toxicity of free extract and extract-loaded in PLGA and PCL NPs for skin application was evaluated by the MTT assay in the human keratinocyte cell line, HaCaT. These cells were seeded in a 96-well plate at a concentration of 5×10^4^ cells/mL in DMEM with high-glucose (4500 mg/L), supplemented with 10% of FBS, and 100 IU/mL of penicillin and 100 µg/mL of streptomycin [(Pen/Strep, 1%, *v*/*v*)] (complete medium), in a humidified chamber at 37 °C in a 5% CO_2_ atmosphere, and allowed to adhere for 24 h [25]. After that time, the medium was removed and extracts at concentrations levels ranging from 2.11–33.75 µg of rutin/mL in free form or loaded in NPs were added to the HaCaT cells. After 48 h of incubation, the medium was discarded and the cells were washed 2 times with PBS (USP41). Then, 50 μL of MTT at 0.5 mg/mL (*p*/*v*) in incomplete medium was added to the cells and the plates were incubated for 4 h at 37 °C in a 5% CO_2_ atmosphere. After the incubation time, 100 μL of DMSO were added to each well for solubilizing the formazan crystals. Then, the Abs was measured, and cell viability was calculated using Equation (7):

(7) $$Cell\;Viability\;\left(\%\right)=\;\frac{Abs_{t}}{Abs_{c}}\times 100$$

where *Abs_t_* is the absorbance of tested samples and *Abs_c_* is the absorbance of control cells (incubated with complete medium). A total of three independent experiments, with six replicates per condition, were conducted.

- Cell Viability of Human Fibroblast Cells

HFF cells were cultured in Glutamax™ IMDM and supplemented with heat-inactivated FBS (10%, *v*/*v*), 100 units/mL of penicillin and 100 µg/mL of streptomycin. All experiments were performed in culture medium supplemented only with 0.5% of FBS without any antibiotic [26]. The cells were seeded at 1 × 10^5^ cells/mL of density in 96-well plates and allowed to grow for one week (to assure the complete confluence), with medium renewal every 24 h. The cells were cultured in a humidified atmosphere at 37 °C with 5% CO_2_. After that time, the cells were incubated with different concentrations of extract in free form or loaded in PLGA and PCL NPs, diluted in Glutamax™ IMDM and FBS (0.5%). The tested concentrations ranged from 1.81 to 29.02 μg for rutin/mL and according to the previous study [6]. After 48 h of incubation, the cell proliferation was measured using PrestoBlue^®^ Cell Viability Reagent and the fluorescence values were analyzed in a Microplate Fluorimeter FL× 800 (Bioteck Instruments, Winooski, VT, USA) (excitation and emission wavelengths of 580 nm and 595 nm, respectively). The cell’s viability was calculated in relation to control with complete medium (without samples). At least three independent experiments were made using three replicates. The fluorescence was measured, and the cell’s viability was calculated using Equation (8):

(8) $$Cell\;Viability\;\left(\%\right)=\;\frac{Flu_{t}}{Flu_{c}}\times 100$$

where *Flu_t_* is the fluorescence of tested samples and *Flu_c_* is the fluorescence of control cells (incubated with complete medium). A total of three independent experiments, with six replicates per condition, were conducted.

#### 2.2.14. In Vitro and In Vivo Anti-Inflammatory Activity

The anti-inflammatory activity (AIA) was assessed in vitro and in vivo according to the following procedures:

- In Vitro Anti-Inflammatory Activity

In vitro AIA was determined through reduction of nitric oxide (NO) production in RAW264.7 cells. The RAW264.7 cells (ATCC^®^ SC-6003™) were used in DMEM culture medium supplemented with 10% of heat-inactivated FBS, 1% of L-glutamine (2 mM), and 1% of Pen (50 U/mL)/Strep (50 μg/mL), at 37 °C in a humidified atmosphere and 5% CO_2_. The cells were seeded in 96-well tissue plates until 80% of confluence. Then, different samples, namely free extract (35 µg/mL) and extract-loaded NPs (35 µg/mL), were incubated with cells (density 2.5 × 10^5^ cells/well) in a 96-well-plate containing 100 ng/mL of lipopolysaccharide (LPS), for 24 h. An equivalent concentration of empty NPs was also tested. Cellular viability was determined by the MTT assay, in relation to a control with the vehicle (0.5%, DMSO), and according to Equation (7) as described elsewhere [13].

Free nitrites in culture medium were measured by spectroscopy at 562 nm using the *Griess* method. The amount of nitrite was calculated from a NaNO_2_ standard curve (concentration range 0–100 µM). The equation was y = 0.0114x + 0.0578 with R^2^ of 0.9962. The *NO production* (%) of the tested samples [NO^2−^] in relation to the negative control [NO^2−^] was calculated using Equation (9):

(9) $$NO\;Production\;\left(\%\right)=\;\frac{\left[{\mathrm{NO}}^{2-}\right]t\;}{\left[{\mathrm{NO}}^{2-}\right]c\;}\times 100$$

The *NO inhibition* (%) was then determined by using Equation (10):

(10) NO Inhibition (%)= 100− NO Production 

- In Vivo Anti-Inflammatory Activity

The carrageenan paw oedema model in rats is one of the most used tools to study the acute local inflammation [13,27]. In this assay, animals were randomly allocated into different groups (each group with *n* = 8, except positive control *n* = 6) and were anesthetized by intraperitoneal injection with a mixture of ketamine (75 mg/kg) and medetomidine (0.50 mg/kg). The AIA of the extract in the free form or loaded in NPs was evaluated after topical administration. Each formulation was applied in the right hind paw of each animal (200 µL). The extract concentration applied (free extract and extract-loaded NPs) was 1.0 mg/kg body weight. In the negative control group, a placebo Carbopol^®^ 940 gel was applied. In the case of the positive control group, a dose of 1.0 mg/kg body weight commercial diclofenac sodium-containing formulation was used. Based on the literature, the Carbopol^®^ 940 gel was prepared with some modifications [28]. In brief, 500 mg of Carbopol^®^ 940 were dispersed in water under magnetic stirring (400 rpm). After the polymer was fully solubilized, 0.2% of methyl 4-hydroxybenzoate and 0.02% of propyl-4-hydroxybenzoate were added under constant stirring. Finally, 0.2 g of NaOH was added under magnetic stirring, promoting the gelation of Carbopol^®^ 940. Both NPs were incorporated into the gels, as well as in the free extract. Incorporation of extract-loaded PLGA NPs was performed by adding 2.1 mL per mL of gel; the incorporation of extract-loaded PCL NPs was performed by adding 1.65 mL per mL of gel. Finally, 1.5 mg per mL of gel were added in the free extract. The concentrations of free extract and extract-loaded NPs administered were based on the EE obtained for each NP and on the maximum concentration of the extract previously obtained [6].

**Figure 1:**
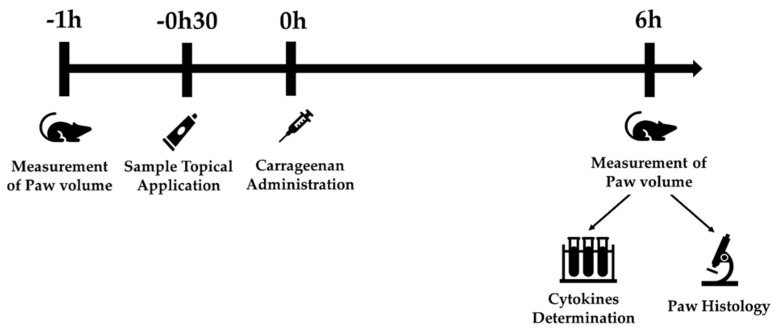
The experimental design of carrageenan oedema model.

The experimental design was performed according to Figure 1. Paw oedema was induced by a single subplantar injection of 0.1 mL of a λ-carrageenan (1%, *w*/*v*) sterile saline solution 30 min after topical treatment in the rats’ right hind paw. The paws’ volume was measured before the sample topical application and carrageenan injection (V_0_ or basal volume) and 6 h later (V_6_) using a plethysmometer (Ugo Basile, Gemonio (VA), Italy).

Oedema inhibition (%) was determined according to Equation (11):

(11) $$Oedema\;Inhibition\;\left(\%\right)=\;\frac{\left|{\left(V_{6}-\;V_{0}\right)}_{negative\;control}\right|-\;{\left(V_{6}-\;V_{0}\right)}_{animal}}{\left|{\left(V_{6}-\;V_{0}\right)}_{negative\;control}\right|}\;\times 100$$

Six hours after the carrageenan injection, the paws’ volume was measured again and right after animals were anesthetized with isoflurane. Blood was collected by cardiac puncture for K_2_ EDTA tubes. Specimens of paws were excised and fixed in 10% buffered formalin. After fixation, they were decalcified using 7% Ethylenediamine tetraacetic acid (EDTA) and then processed for routine hematoxylin-eosin (H&E) staining. Slides were analyzed under an Olympus BX51 Microscope (Olympus Corporation, Tokyo, Japan) and whole-slide scanning was performed using a NanoZoomer-SQ Digital slide scanner C13140-01 (Hamamatsu Photonics, Shizuoka, Japan). Images were taken using the NDP. Viewer software (Hamamatsu Photonics, Shizuoka, Japan). Plasma samples were tested to quantify cytokines as interleukins (IL-6 and IL-10) and tumor necrosis factor-alpha (TNF-α), all expressed as pg/mL. The cytokine levels were measured spectrophotometrically at 450 nm (ELISA kit Quantikine, Hycult Biotechnology, Wayne, PA, USA).

#### 2.2.15. Statistical Analysis

The results are expressed as mean ± standard deviation (S.D.) for the in vitro studies. For biological assays, the results are expressed as mean ± standard error of the mean (S.E.M.). The significance of differences was assessed by using One-Way ANOVA for multiple comparisons between all the samples, using the Graph Prisma Version 5.03 (GraphPad Software, San Diego, CA, USA). The differences were considered significant when *p* < 0.05. Two-Way ANOVA for multiple comparisons between all the samples was also used when comparing results over time.

## 3. Results and Discussion

### 3.1. Quantification of Major Compounds and the Residual Quantity of Methanol

**Figure 2:**
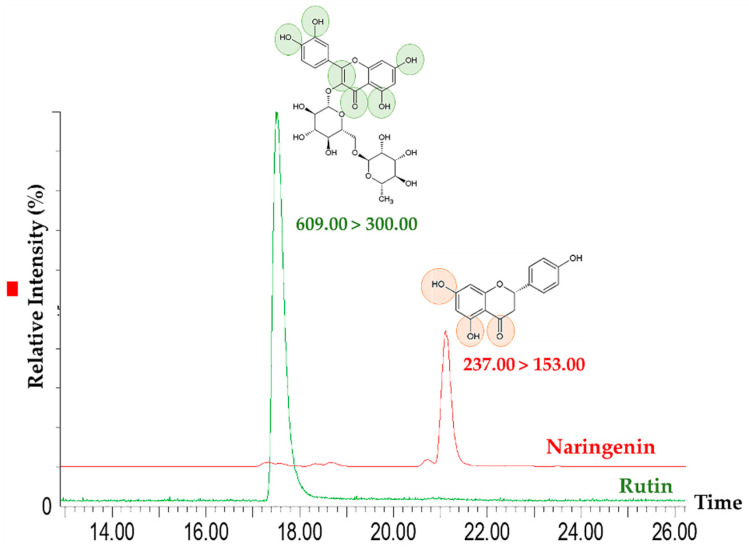
MRM chromatograms of rutin and naringenin in elderflowers’ methanolic extract.

In recent last years, a re-emerging interest in natural products for industrial purposes has been observed due to their reputable sources of new active pharmaceutical ingredients [1]. *S. nigra* L., for example, has been extensively used in traditional medicine, particularly in Europe, where it has been officially recognized as a potential bioactive ingredient by the European Union. Due to its properties in several supplements and nutraceutical products, it is now commercially available in markets in different countries [3,29]. However, the biological activities of elderflowers have been demonstrated mostly in non-clinical studies [6]. Therefore, the aim of the studies performed by our group was to provide a scientific support and validation to the use of *S. nigra* L. [5]. In a previous study, several compounds were identified in the elderflowers’ methanolic extract, including rutin, isoquercetin, isorhamnetin-3-*O*-glucoside, naringenin, quercetin-4-*O*-glucoside, isorhamnetin-3-rutinoside, luteolin-7-*O*-glucoside, malic acid, and eriodictyol [6]. In the same work, rutin and naringenin were identified as the major phenolic compounds in extract (Figure 2) [6]. These compounds were quantified at 74.93 ± 17.00 mg/g and 31.70 ± 0.14 mg/g of extract, respectively. The quantification of naringenin was done mainly due to the association of this flavonoid to the Coll inhibition [30], as previously reported [6].

**Figure 3:**
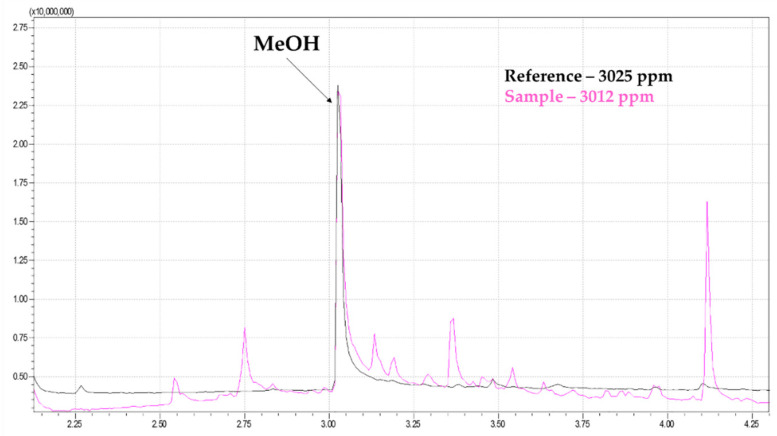
Comparison of chromatographic profile of a MeOH reference with 3025 ppm and a sample of extract as generated by SPME-GC-MS.

The analysis of volatile compounds, particularly MeOH, was performed to quantify the residual amount of the solvent used in the extraction method. The residual MeOH concentration in the extract was 3012 ppm, as shown in Figure 3. The ICH Q3C (R6) classify the MeOH as a Class II solvent and it established the limit of 3000 ppm per day [31]. The results revealed solvent levels just around this value, with no statistical difference when compared to the guideline’s recommendation. Assuming that the same dose is safely used in the in vivo assays to a 70 kg human adult, the amount of methanol would be around 105 ppm per dosage, which is below the maximum dosage recommeded per day.

### 3.2. Characterization of Nanoparticles

Nanotechnology was used to increase the stability of active compounds, to modulate their release, as well as to improve the solubility of poorly water-soluble compounds [1,32,33]. Thus, two different polymeric NPs for skin delivery were selected: PLGA and PCL NPs polymers [12,34].

**Figure 4:**
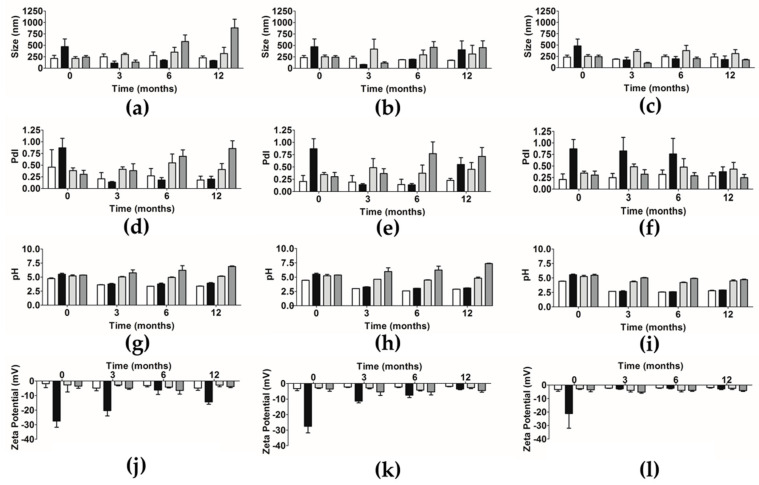
Mean size, PdI, pH, and zeta potential of empty PLGA NPs (white columns), empty PCL NPs (light grey columns), extract-loaded PLGA NPs (black columns) and extract-loaded PCL NPs (dark grey columns) suspension over time: mean size (**a**–**c**), PdI (**d**–**f**), pH (**g**–**i**), and zeta potential (**j**–**l**). (**a**) size at RC, (**b**) size at RT, (**c**) size at AC, (**d**) PdI at RC, (**e**) PdI at RT, (**f**) PdI at AC, (**g**) pH at RC, (**h**) pH at RT, (**i**) pH at AC, (**j**) zeta potential at RC, (**k**) zeta potential at RT, and (**l**) zeta potential at AC (mean ± S.D., *n* = 9). Refrigerated Conditions—RC, Room Temperature—RT, and Accelerated Conditions—AC.

Figure 4 shows the results of mean size, PdI, pH, and zeta potential of NPs suspension over time and at different storage temperatures: Refrigerated Conditions (RC), Room Temperature (RT), and Accelerated Conditions (AC).

The DLS analysis demonstrated that empty PLGA and PCL NPs were stable over time for all parameters in the three different tested temperatures. After loading, the mean size of extract-loaded PLGA NPs at RC and AC decreased from month zero (471.3 ± 169.9 and 480.0 ± 150.8 nm) to month 12 (164.2 ± 6.4 and 180.8 ± 78.0 nm). However, at RT this effect was not observed. In this sense, the results suggest that extract-loaded PLGA NPs are stable over time, mainly at RT. An increase of size after extract encapsulation was observed; however, this change is less pronounced over time for all temperatures. In the case of extract-loaded PCL NPs, at RC and RT, an increase of size values from 242.7 ± 33.7 nm and 245.5 ± 35.3 nm (0 months) to 882.1 ± 189.4 nm and 451.6 ± 147.9 nm (12 months), respectively, was observed. However, at AC, they presented similar values over time from 192.7 ± 83.8 nm (0 months) to 173.6 ± 12.2 nm (12 months). A slight increase of size was observed after extract encapsulation; however, this increase is less pronounced over time for all temperatures. Concerning the extract-loaded PCL NPs, the influence of temperature on NPs means that size was temperature-dependent, decreasing with the increase of the temperature. To conclude, the extract’s encapsulation caused an increase in the particle’s size, which is in line with what the literature mentions for other or similar compounds of the same type of NPs [5,13]. However, PCL NPs presented similar sizes, with only a slight decrease, which is also in line with the literature, for this type of NPs [12]. In contrast with empty NPs, the extract-loaded PLGA NPs revealed a decrease in size at the different temperatures tested over 12 months, while the extract-loaded PCL NPs presented an increase in size for RC and RT and a decrease in size at AC. The decrease of PLGA NPs’ can be associated to the intrinsic nature of the polymer; PCL presents a higher hydrophobicity that can also contribute to this change [35]. Moreover, as it was verified by the TEM and AFM analysis, the PCL NPs presented an higher agglomeration tendency when compared to the PLGA NPs, which is also supported by the literature [36]. 

Particle’s size is considered as a key factor for skin delivery, although the ideal size of NPs is still not consensual. According to the literature, particles between 300 and 500 nm can penetrate through intact skin [37]. However, the administration of NPs can be affected by skin barrier changes such as scrapes, wounds, dermatitis conditions, etc. Moreover, factors, such as age, gender, skin thickness and vascularity, density of hair follicles, mechanical trauma (integrity of the skin), and diseases can also have an impact on topical administration [1].

In addition to the particle’s size, it is also important to have monodisperse batches in order to better predict the stability, as well as the in vivo behavior of any nanoproduct. The PdI values for extract-loaded PLGA NPs presented the same statistical difference for the three temperature conditions tested. In general, they presented monodisperse populations over time, except at time 0 for the three temperatures and after 12 months under RT. Furthermore, under AC the results suggest that those NPs present higher polydisperse populations which were stable over time, mainly at RT. Although, after encapsulation, an increase of the PdI value was observed, this improvement was less pronounced over time for RC and RT. The extract-loaded PCL NPs presented polydisperse populations, mainly at RC and RT. The major difference between the values of time 0 and time 12 was verified at RT, followed by RC and finally the AC. These results suggest that these NPs are stable over time, mainly at AC, where the values of PdI are closer to monodisperse populations (<0.25, as previously mentioned). The extract-loaded PCL NPs presented a decrease of PdI, that it was only maintained under AC. To conclude, there was a reduction of PdI of extract-loaded PLGA NPs, while extract-loaded PCL NPs exhibited an increase of PdI at RC and RT, with a decrease at AC. This means that PLGA NPs might be more stable. The addition of extract to the PLGA NPs displayed an increase in size, which is in line with the literature for other similar compounds in the same type of NPs [5,13]. However, concerning the PCL NPs, they presented similar sizes with a slight decrease, which is also in line with the literature [12]. Nevertheless, the overall assessment of PdI confirms the physical stability of the formulations throughout the study. The addition of extract to the formulation resulted in an initial decrease of PdI.

pH was also assessed as a good stability prediction parameter. Concerning pH values, the extract-loaded PLGA NPs’ suspensions presented the same statistical difference of *p* < 0.001 over time, and at the different analyzed temperatures, which suggests that the variation of temperature has not influenced pH values. However, the major difference between the values at time 0 and time 12 was observed at AC, followed by RT and finally by RC. In this sense, the results suggest that the extract-loaded PLGA NPs are stable over time, mainly at RC. After encapsulation, an increase of pH value of NPs suspension was observed, which was maintained over time, probably due to the PLGA hydrolysis to lactic and glycolic acids. The extract-loaded PCL NPs presented the same statistical difference over time at the three different temperatures, which suggests that the change of temperature was not influenced by the statistical difference of pH values of NPs suspension. However, the major difference between the values at time 0 and time 12 was verified at RT, followed by RC and AC. From that point of view, these results suggest that the extract-loaded PCL NPs are stable over time, mainly at AC. Similarly to what was observed in PLGA NPs, the extract’s encapsulation into the PCL NPs increased the pH values of the respective suspension. Both values observed throughout the study are compatible to skin application [32], where the ideal range of pH is 3.5–5.0 [38,39]. After encapsulation, the extract-loaded formulation displayed an increase of pH value, which is in line with the literature in the same type of NPs, PLGA [5] and PCL [12].

The zeta potential analysis revealed that all the extract-loaded NPs (PLGA and PCL) were negatively charged. Both are stable over time, mainly at RC and AC, respectively. Zeta potential analysis suggests that an increase of temperature resulted in less negative charge of extract-loaded PLGA NPs over time, while the surface charge of extract-loaded PCL NPs was not apparently affected by temperature.

**Figure 5:**
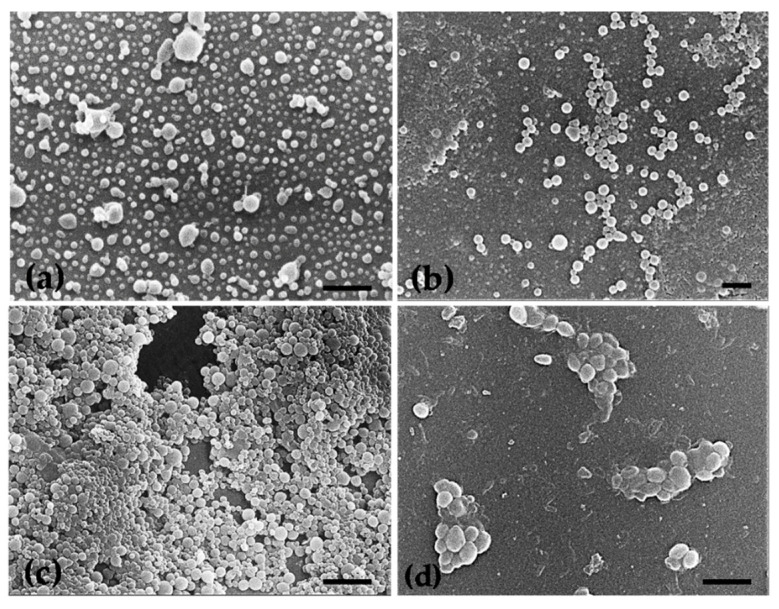
SEM micrographs showing the morphology of NPs. (**a**) Empty PLGA NPs, (**b**) Extract-loaded PLGA NPs, (**c**) Empty PCL NPs and (**d**) Extract-loaded PCL NPs. Scale bars = 2 µm (**a, b**), 10 µm (**c**,**d**).

Morphology is also a key factor for skin permeation [40]. The extract-loaded NPs (both PLGA and PCL) or empty NPs observed by SEM presented a general spherical shape, a smooth surface, and a size range below ≈471 nm for extract-loaded PLGA NPs and ≈243 nm for extract-loaded PCL NPs (Figure 5), confirming DLS data. Following the extract’s encapsulation in PLGA NPs, the morphology did not changed. In the case of PCL NPs, a different density of particles between empty and extract-loaded PCL NPs was observed (Figure 5c,d, respectively).

**Figure 6:**
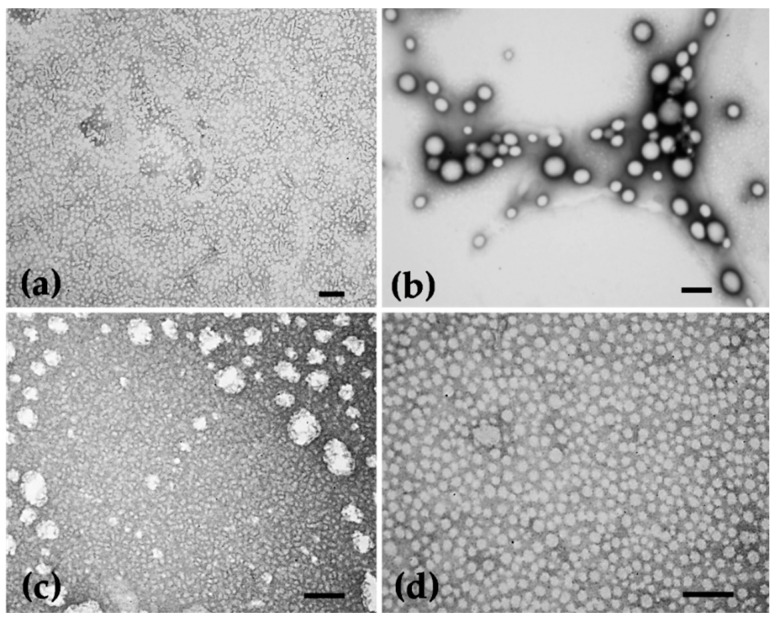
TEM micrographs showing the morphology of NPs. (**a**) Empty PLGA NPs, (**b**) Extract-loaded PLGA NPs, (**c**) Empty PCL NPs and (**d**) Extract-loaded PCL NPs. Scale bars = 0.2 µm (**a**,**b**,**c**), 0.1 µm (**d**).

TEM observations confirm the particles’ spherical shape, their nanometric dimensions and their uniform size distribution (Figure 6), which is in line with to the results obtained by the DLS method.

**Figure 7:**
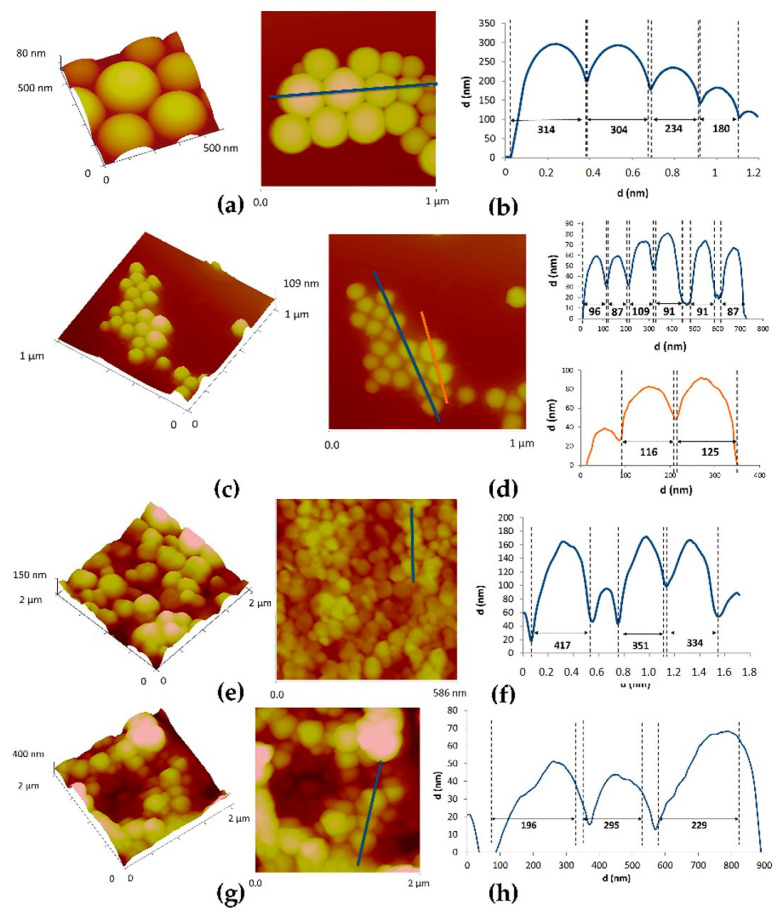
AFM topographic images of NPs under study. (**a**,**b**) Empty PLGA NPs, (**c**,**d**) Extract-loaded PLGA NPs, (**e**,**f**) Empty PCL NPs, (**g**,**h**) Extract-loaded PCL NPs. 3D images (**a**,**c**,**e**,**g**) and cross section analysis (**b**,**d**,**f**,**h**).

Figure 7 shows the AFM images obtained for the different NPs, as well as their corresponding section profiles. The AFM analysis showed a mean particle size of 255 ± 55 nm for empty PLGA NPs, a smaller particle size after loading it with 99 ± 20 nm. PCL NPs had a larger size in comparison with PLGA NPs (367 ± 44 nm for empty PCL NPs and 243 ± 62 nm for extract-loaded PCL NPs).

The spherical shape was reported as the ideal form for a fast endocytosis [41]. In the current study, all types of NPs showed a spherical shape when observed by SEM, TEM, and AFM.

The encapsulation efficiency (EE) in NPs was determined in terms of rutin concentration. The value was very similar for PLGA NPs (75.90 ± 31.14%) and PCL NPs (86.31 ± 25.21%).

**Figure 8:**
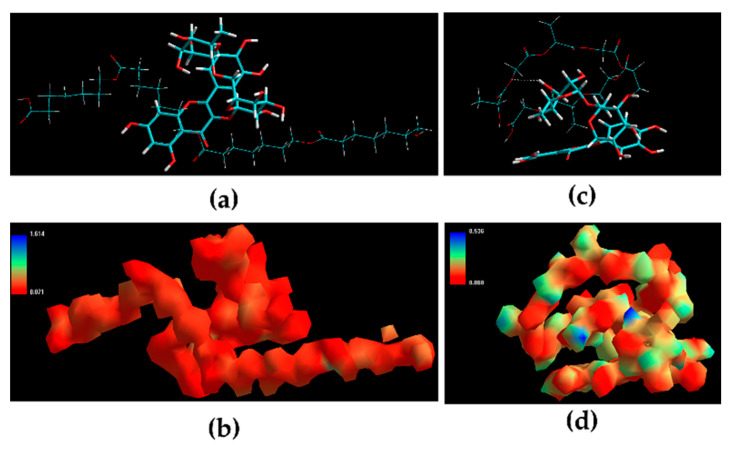
Representation of (**a**,**c**) geometrical preferences (**b**,**d**) electrostatic molecular graph after molecular mechanics simulations in vacuum (**a**,**b**) PCL-Rutin (**c**,**d**) PLGA-Rutin [Colour code for elements: C = cyan; H = white; O = red; N = blue]. Rutin: tube rendering; PCL: stick rendering; PLGA: stick rendering.

In order to clarify the EE values, the complex of rutin and the polymers of NPs (PLGA and PCL) were analyzed. Table 1 presents the inherent molecular energy attributes for various biomolecular complexes, while Figure 8 shows the corresponding geometrical positions, which confirm a relation between the rutin and the polymers used in each NPs (PLGA and PCL).

The extract was successfully entrapped in both carriers. This observation was supported by analyzing the rutin-polymer complexes, which suggested that rutin-PLGA was the most stable molecular structure (∆E ≈ −20 kcal/mol). The rutin-PCL complex also showed considerable energy minimization (∆E ≈ −18 kcal/mol) compared to PLGA. The favorable energy stabilization can be attributed to the high encapsulation efficiency of the polymeric NPs. The rutin-polymer complexes demonstrated similar energy stabilizing profile involving non-bonding dihedral and bonding van der Waals (vdW) components. The dihedral energy component confirmed the anti-inflammatory molecule as a good fit for the polymer matrix.

**Figure 9:**
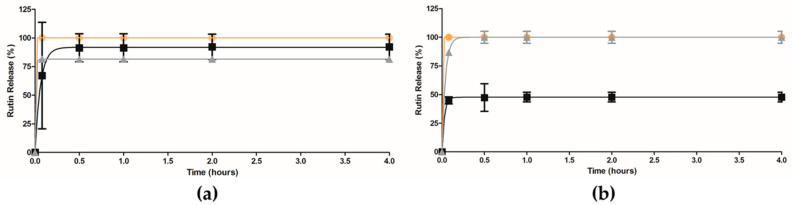
Percentage of the major compound (rutin) release over time in (**a**) PBS at pH 5.5 and (**b**) PBS at pH 7.4: free extract (orange line), extract-loaded PLGA NPs (black line) and PCL NPs (grey line) (mean ± S.D.).

To the best of our knowledge, a comparative release study of the methanolic elderflowers’ extract from two different types of NPs was herein performed for the first time. Two different pH were used in order to simulate the biological conditions: pH 5.5 similar to the pH of the skin [32] and pH 7.4 that corresponds to the biological pH of the blood and interstitial’s fluid [42]. The graphic representation is shown only until 4 h, since the percentage of rutin released was constant up to 24 h (Figure 9).

Rutin of the free extract was immediately dissolved in both buffers. But, after encapsulation, a different release of rutin was observed in both NPs (Figure 9a,b). In comparison to PLGA, the rutin release from PCL NPs was faster, especially in PBS at pH 7.4 (Figure 9b). The results show a higher rutin release from PLGA NPs after 30 min in PBS pH 5.5, whereas in pH 7.4, PCL NPs presents a complete rutin release. The results presented the same behavior for the same type of NPs when berberine was encapsulated, PLGA NPs presented a higher release when exposed to the PBS pH 5.5 than to the pH 7.4 [43]. Another study with PCL NPs and curcumin presented similar results with the ones obtained in the present study, with lower release from PCL NPs in PBS pH 5.5 than in pH 7.4 [44]. Thus, according to our previous results, the encapsulation may be an additional advantage due to the lower release in the first time points when compared with the free extract. Furthermore, these data also are in agreement with the results obtained for rutin-polymers complexes, where a higher energy variation for rutin-PLGA NPs was observed. Moreover, the presence of H-bonding in the rutin-PLGA complex may have led to the retention of the rutin molecule within the PLGA matrix at physiological pH. Another important factor that can be associated with the rutin release is the fact that PCL presents higher hydrophilicity than PLGA [35].

**Figure 10:**
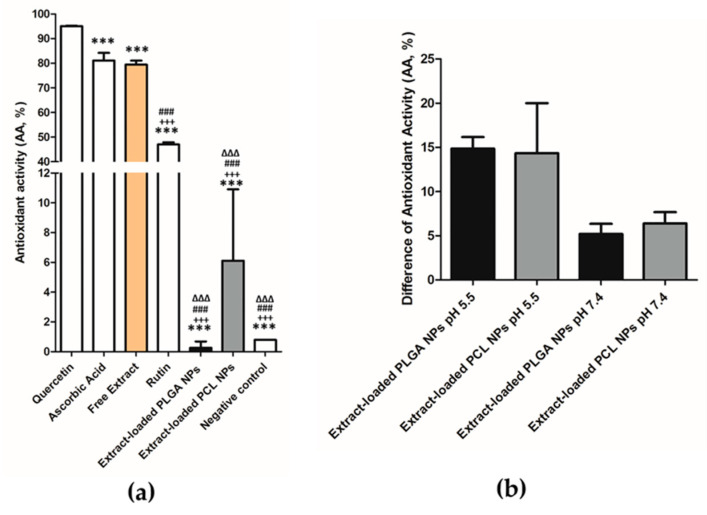
(**a**) AA of quercetin and ascorbic acid (positive controls), free extract, extract-loaded PLGA NPs, extract-loaded PCL NPs, (%, mean ± S.D.; n = 3, *** *p* < 0.001, when compared the quercetin with the other samples, ^+++^ *p* < 0.001, when compared the ascorbic acid with the other samples, ^###^ *p* < 0.001, when compared the free extract with the other samples and ^∆∆∆^ *p* < 0.001, when compared the rutin with the other samples), (**b**) Difference of AA in release medium when compared with respective extract-loaded NPs (%, mean ± S.D.; *n = 3*).

The antioxidant activity (AA) was assessed by three different methods: DPPH, ORAC and HOSC. Concerning DPPH, and according to previous studies performed by our group, this extract presents a high AA, similar to positive controls (Figure 10a). Empty NPs were also tested, revealing no AA.

The free extract presents an AA of 79.5 ± 1.6%, while the ascorbic acid and the quercetin present values of 83.0 ± 3.7% and 95.1 ± 0.2%, respectively. Differences were only observed between the extract and the quercetin (*p* < 0.001). The AA results obtained by DPPH revealed that the extract-loaded NPs presented a significant decrease of the AA in comparison with the free extract. The decrease of AA after encapsulation probably means that the compounds responsible for the AA might not be completely exposed or released, as suggested by the interactions between the complex rutin-polymers (PLGA or PCL), due to the reduced reaction time of this assay (very fast assay ≈30 min). To perform the DPPH method, these radicals have to be easily accessible, which was not verified for the extract-loaded NPs. Afterwards, these systems were analyzed by using the same method, 24 h after incubation in PBS buffer pH 5.5 and 7.4 (Figure 10b). This analysis revealed low values of AA for both NPs, where the extract-loaded PLGA NPs in PBS at pH 5.5 is higher than at pH 7.4 (14.9 ± 1.3% versus 14.3 ± 5.7%), thus corroborating the release assays, where the extract is delivered easier at pH 5.5. The extract-loaded PCL NPs presents similar values at pH 7.4 and at pH 5.5 (6.4 ± 1.3% *versus* 5.1 ± 1.2%), whereas for the free extract an AA lower than 30% was observed under same conditions, while the degraded NPs did not reveal any AA by DPPH.

**Figure 11:**
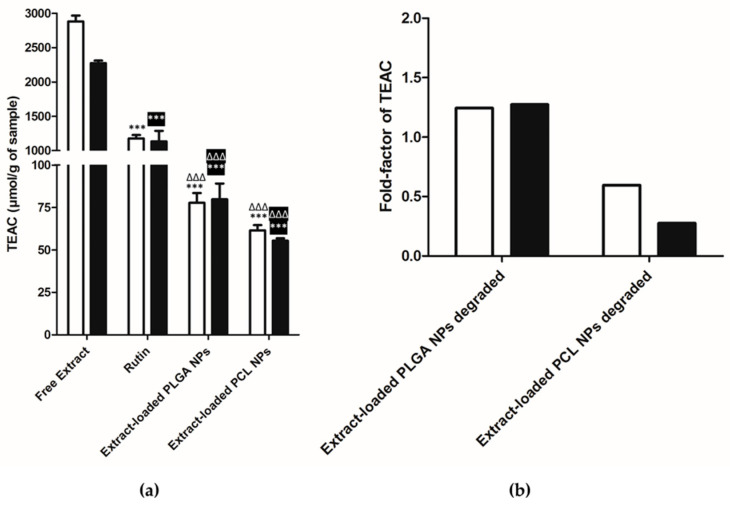
(**a**) Antioxidant activity of free extract, rutin, extract-loaded PLGA NPs and extract-loaded PCL NPs (TEAC (µmol/g of sample, mean ± S.D., *n* ≥ 3, *** *p* < 0.001, when compare free extract with other samples and ^∆∆∆^ *p* < 0.001, when compare rutin with other samples), (**b**) Fold-factor of TEAC when compared with respective extract-loaded NPs, (□) ORAC and (■) HOSC methods.

The other two methods (ORAC and HOSC) were used to get further information about the decrease of AA. The results presented in Figure 11a were obtained by Trolox equivalent antioxidant capacity (TEAC, µmol/g of sample) which means that for free extract is µmol/g of extract, rutin is µmol/g of rutin and for extract-loaded NPs is µmol/g of NPs.

The oxygen radical absorbance capacity (ORAC) and the hydroxyl radical scavenging capacity (HOSC) were used to estimate the AA of extract and extract-loaded NPs. The results suggest that the percentage of rutin in the extract is around 43.82% and 51.22% for ORAC and HOSC, respectively. Values were in line with the previous findings, where rutin was identified as a major compound. But other polyphenols in this extract might be responsible for the remaining TEAC of the extract. The results were the expected due to the extract-loaded NPs, being faster for PLGA NPs than for PCL NPs [45,46]. However, these values are still lower when compared to the free extract, suggesting that the extract is really encapsulated into the NPs, as already mentioned. Concerning the extract-loaded PLGA NPs degraded, there was a decrease of the TEAC when compared to the PLGA NPs, 13.08 ± 4.21% (*n* = 9) and 17.93 ± 9.19% (*n* = 9) for ORAC and HOSC, respectively. Furthermore, the extract-loaded PCL NPs degraded showed an increase of the TEAC when compared to the extract-loaded PCL NPs, 40.07 ± 4.32% (*n* = 4) and 152.36 ± 15.20% (*n* = 6) for ORAC and HOSC, respectively. These differences correspond to the fold-factors presented in Figure 11b.

To conclude, the AA assessed by three different methods showed unexpected and somehow disappointing results. The ORAC method has been widely accepted as a standard tool to measure the AA in the nutraceutical, pharmaceutical, and food industries [19]. Same experimental details may also contribute for this outcome, which does not allow an extensive release of the extract. The extract-loaded NPs displayed different behaviors according to the polymer. Apparently, the compounds responsible for AA were not released from both types of NPs or may have suffered some degradation. Further studies should be conducted in order to elucidate this finding. Nevertheless, the TEAC value was very high after encapsulation, thus corroborating literature and suggesting a potential synergetic effect of the compounds present in the extracts.

The TPC was also assessed and the free extract presented a TPC value of 1605 ± 66 mg gallic acid equivalent (GAE)/L, whereas the extract-loaded PLGA and PCL NPs did not show any polyphenol content using these experimental conditions. This assay is in accordance with the AA determined by the DPPH method.

**Figure 12:**
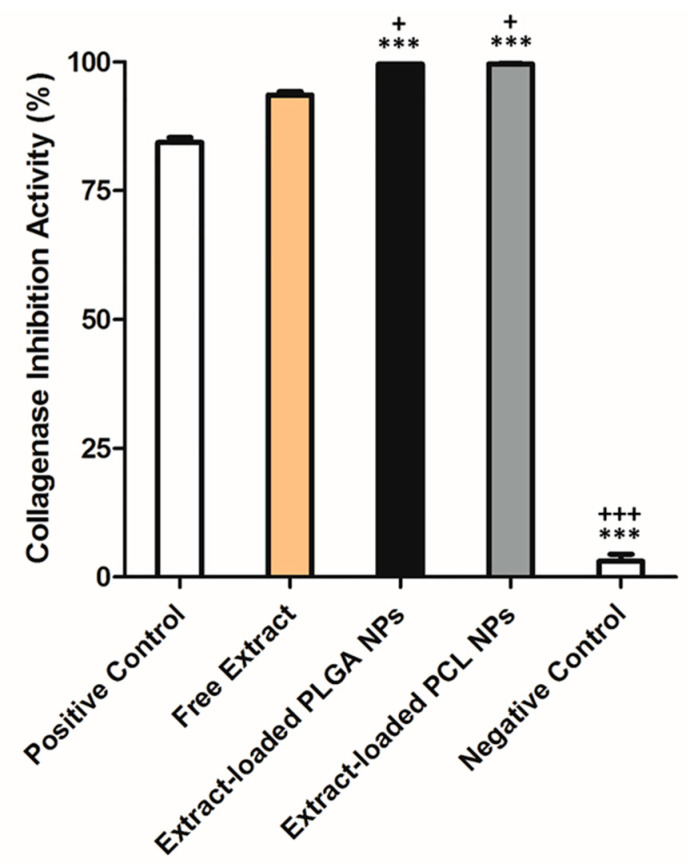
In vitro collagenase inhibition activity of samples (at least mean ± S.D., *n* = 3, *** *p* < 0.001, when compared to positive control and ^+^ *p* < 0.05 and ^+++^ *p* < 0.001, when compared to free extract, respectively).

In vitro Coll inhibition activity results are displayed in Figure 12. The free extract presented the highest Coll inhibition activity, even higher than the positive control group. Similar values were observed for the extract-loaded in PLGA and PCL NPs. A fold factor of 1.06 between the free extract and the extract-loaded NPs was observed, suggesting a potential synergistic effect between the extract with the polymers (PLGA and PCL). Coll inhibition activity increased around 10–15% after encapsulation in both types of NPs. Coll is involved in some skin disorders associated to the degradation of collagen-rich extracellular matrix (ECM), as in tumor invasion [6]. Therefore, Coll inhibition might be an important factor to prevent or treat such diseases, where some of the phytocompounds identified in this extract, e.g., naringenin, may play an important role [30].

### 3.3. In Vitro Safety Assessment

**Figure 13:**
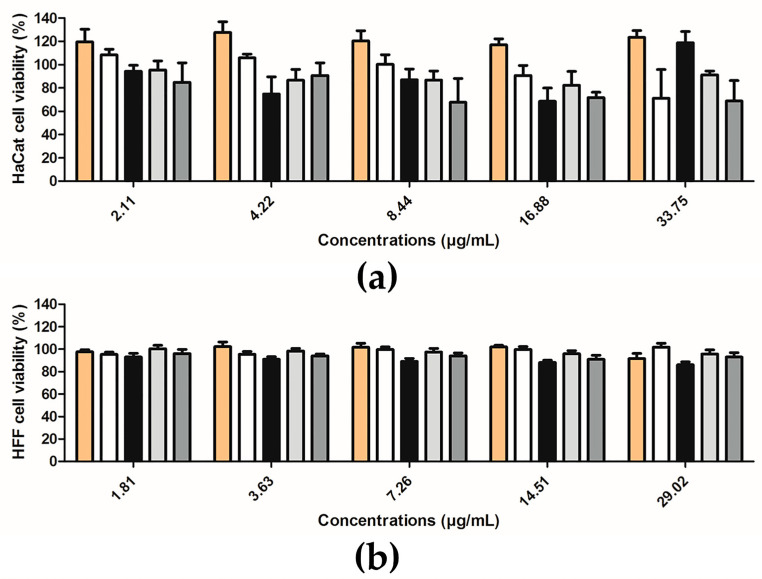
Cell viability (%) of (**a**) HaCaT and (**b**) HFF cells after 48 h incubation (%, mean ± S.D.; *n* > 4): free extract (orange columns), empty PLGA NPs (white columns), extract-loaded PLGA NPs (black columns), empty PCL NPs (light grey columns) and extract-loaded PCL NPs (dark grey columns).

One key parameter in pharmaceutical development is the safety of the formulations. In this work, two relevant cell lines were studied: HaCaT cells and HFF cells. These two cell lines showed that both polymeric formulations were safe. Viability studies of HaCaT cells were performed for all samples, in the concentration range of 2.11–33.75 µg of rutin/mL (Figure 13a). No change in cell viability was observed. The same observation occurred with HFF cells for a range of concentrations of 1.81–29.02 μg for rutin/mL (Figure 13b). The selection of these two type of cells was based on each cell’s properties and functions. HaCaT cells are a spontaneously transformed keratinocytes cell line from adult human skin with high capacity to proliferate in vitro [47]. HaCaT cells are a nontumorigenic cell line [48,49,50], representing 95% of epidermal cells [51]. This cell line is more sensible than skin [52]. HFF cells are another type of cells which are present in the skin, more specifically in dermis [32]. They are responsible for the production of compounds in the connective tissue (mast cells and macrophages), which are related to the immune and inflammatory responses [32].

### 3.4. Efficacy of Nanocarriers

The AIA of extract was tested both in vitro and in vivo. In vitro pro-AIA based on NO inhibition was registered in Table 2. According to these results, the extract-loaded PLGA NPs led to a lower value of NO inhibition when compared to the extract-loaded PCL NPs. Higher concentrations of both NPs carriers cannot be tested due to limitations of the method, i.e., increasing NPs’ concentration, turbidmetry of the suspension also increased and no reliable values were obtained by spectrophotometry. In any case, these results suggest that the encapsulation appears to be an advantage, especially for extract-loaded in PLGA and PCL NPs, as demonstrated by statistical differences in comparison to the free extract.

A comparative in vivo study using two different types of NPs for methanolic elderflowers extracts was reported for the first time. The results, concerning the in vivo AIA of the extract in the different formulations compared with controls, are presented in Table 3.

**Figure 14:**
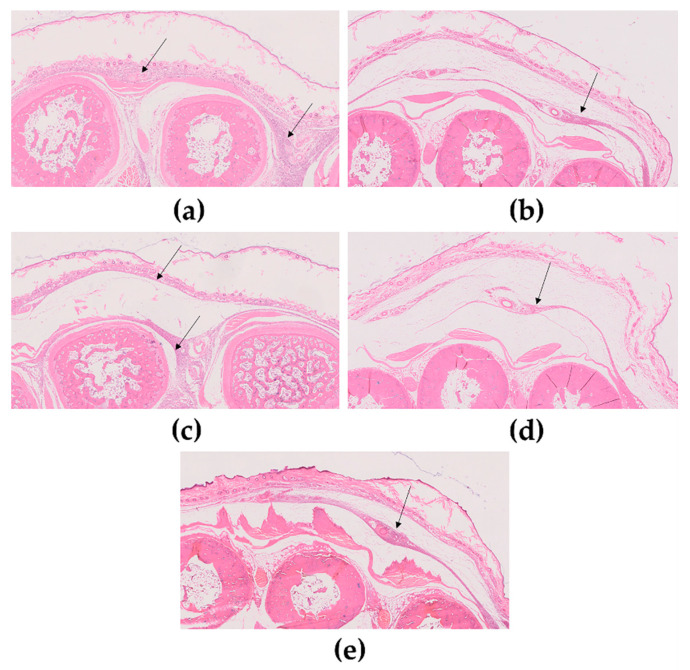
Representative histological images of rat’s paws (2.5×, H&E) after topical administration. (**a**) Negative Control; (**b**) Positive Control; (**c**) Free extract; (**d**) Extract-loaded PLGA NPs; (**e**) Extract-loaded PCL NPs. Arrows show the inflammation area.

The model used/applied created an oedema in rats’ paws. The positive control has an inhibition of oedema. The highest inhibition of extract-loaded NPs was observed for PLGA NPs, presenting more 23% of inhibition, when compared to the free extract. Moreover, the encapsulation into PLGA NPs presented a better result than the same extract into PCL NPs. Histologically, corresponding results in similar magnitude in the dorsal face of the paws were observed. Extract-loaded PLGA NPs paws showed lower oedema and fewer inflammatory infiltrates (Figure 14d, arrows) in comparison with the other groups. The effect of the free extract (Figure 14c) was slight milder than in the negative control (Figure 14a). Considering the biochemical analysis, the results are displayed in Table 4. It is important to note that the values obtained for the different parameters evaluated in the plasma of the rats for each group are in line with the oedema inhibition and histological results. Lower TNF-α and IL-6 values were observed for rats treated with diclofenac (positive control), followed by extract-loaded PLGA NPs, extract-loaded PCL NPs and, finally, the free extract. Regarding IL-10, higher values were observed for the positive control group, followed by extract-loaded PLGA NPs and, finally, by the extract-loaded PCL NPs. However, the free extract presented a decrease of the IL-10 concentration.

The in vitro AIA revealed an increase of NO reduction after extract nanoencapsulation, mainly into the PCL NPs, whereas in the in vivo AIA studies, the extract’s administration revealed higher AIA with PLGA NPs. This event can be associated to the highest release of rutin from PLGA NPs than from PCL NPs at PBS pH 5.5, the pH of the skin, which can contribute to a higher permeation of rutin with a consequent increase of paw oedema inhibition. This last observation was also supported by histological analyses, where the cytokines levels of extract-loaded PLGA NPs led to similar results of IL-6, IL-10 and TNF-α in comparison to the positive control (diclofenac). According to the literature, there are some compounds that can inhibit the expression or activity of inducible nitric oxide synthase (iNOS) and they have AIA properties [53]. Some potential compounds are flavonoids and other polyphenols, which are involved in suppression of the cytokine’s production [54]. They also seem to present a protective effect against inflammation, reducing the severity of the inflammatory process and regulating the expression of pro-inflammatory cytokines [54]. The extract of elderflowers used in this work presented a rich composition of phytocompounds including the flavonoids rutin and naringenin, as mentioned above. Rutin and naringenin probably might be responsible for the suppression of iNOS protein through the inhibition of nuclear factor-kB (NF-kB) [53,55]. Naringenin was also reported as having mRNA expression and NO production in activated macrophages in a dose-dependent manner [53]. Flavonoids present AIA, acting in the reduction of eicosanoids production through inhibition of the phospholipase A2 (PLA2), cyclooxygenases (COX), and lipoxygenases (LOX) activities, as well as inhibiting phosphodiesterases, protein kinases, histamine releasing, and modulators of the transcription of genes, or due to their AA [54]. As the exact mechanism is still under research, the authors hypothesized that the reported biological activities probably result from a synergistic effect of several extract components with an impact on AIA. This hypotesis is also supported by previous references. Nikfarjam et al. reported that rutin inhibits the TNF-α production in human peripheral blood neutrophils [56,57]. Rutin is also associated to a reduction of IL-6 expression, a reduction of TNF-α [56] and an increase of IL-10 concentration [58]. Remarkably, in the current study, AIA was increased after extract encapsulation in PLGA NPs, which was corroborated by IL-6 and TNF-α levels. Compared to positive control, diclofenac is generally associated to a decrease of IL-6 concentration, a decrease of TNF-α, and to an increase of IL-10 concentration [59,60,61]. In addition, this study also suggests that the mechanism responsible for the AIA of the nanoencapsulated methanolic *S. nigra* extract is not only related to the pro-inflammatory cytokines [6], but also based on the anti-inflammatory cytokines’ production.

## 4. Conclusions and Perspectives

Natural products and their derivatives have been recognized for many years as a source of therapeutic agents that can be less toxic and more effective. It has been estimated that nearly 75,000 species of higher plants exist on the earth, and only 10% have been used in traditional medicine. Only 1 to 5% have been studied scientifically and are known to have therapeutic value.

For the first time, the current study provides a scientific evidence of AIA and Coll inhibition properties of a methanolic extract obtained from *S. nigra* L. flowers. This effect was even more noticeable after encapsulation in PLGA NPs. Both in vitro and in vivo methodologies supported the potential topical use of methanolic extract of *S. nigra* L. obtained from flowers. Comparing the two polymers used as carriers, PLGA NPs displayed a higher value of in vivo AIAby topical route, reaching a mean value of 61% of inhibition of oedema. Similar values of IL-6 and IL-10 were observed bewteen extract-loaded PLGA NPs and the positive control, a commercial formulation of diclofenac. This polymeric carrier also showed a high Coll inhibition activity. On the other hand, PLGA NPs did not cause cytotoxicity in HaCaT and HFF cell lines.

More detailed studies of the AIA should be performed to identify the exact mechanism associated to this biological activity, as well as to verify if the developed system in this work can be applied in a chronic model of inflammation. Overall, the obtained results could certainly attract interest in extract-loaded PLGA NPs as a potential nanoproduct in a very near future.

## Figures and Tables

**Table 1:** Inherent energy attributes of polymer-rutin complexes calculated using static-lattice atomistic simulations (SLAS) in vacuum.

Energy	Rutin	PCL	PCL-Rutin	ΔE ^1^	PLGA	PLGA-Rutin	ΔE ^1^
Total ^2^	23.09	6.41	11.47	−18.04 ^7^	2.64	5.37	−20.36 ^7^
Bond ^3^	1.16	0.34	1.41	−0.09 ^7^	0.29	1.64	0.19 ^8^
Angle ^4^	7.57	1.33	9.14	0.24 ^8^	2.53	13.55	3.44 ^8^
Dihed ^5^	14.32	0.05	8.63	−5.74 ^7^	0.89	14.72	−0.49 ^7^
vdW ^6^	0.04	4.69	−7.71	−12.45 ^7^	−1.06	−24.12	−23.18 ^7^

^1^ ΔE_(A/B)_ = E_(A/B)—_[E_(A)_ + E_(B)_]; ^2^ total steric energy for an optimized structure; ^3^ bond stretching contributions; ^4^ bond angle contributions; ^5^ torsional contribution arising from deviations from optimum dihedral angles; ^6^ van der Waals interactions; ^7^ values represent the structure stabilizing contribution; and ^8^ values represent the structure destabilizing contribution.

**Table 2:** In vitro AIA: NO inhibition values relative to LPS-stimulated RAW264.7 macrophages incubated with free extract and with extract-loaded NPs (mean ± S.E.M., *n* = 6, *** *p* < 0.001, when compared to the free extract).

Samples	Concentration	NO Inhibition (%)
Free Extract	35 µg/mL	23.6 ± 1.8
Extract-loaded PLGA NPs	35 µg/mL	43.5 ± 4.0 ***
Extract-loaded PCL NPs	35 µg/mL	55.3 ± 1.8 ***
Negative control (DMSO)	0.5%	0.0 ± 0.0

**Table 3:** AIA of topically applied extract formulations on the carrageenan-induced rats’ hind paw oedema model (mean ± S.E.M, *n* = 5, unless otherwise indicated; a—*n* = 6; b—*n* = 8, * p < 0.05 and *** p < 0.001, when compared to negative control).

Group	Formulations	Dose (mg/kg)	Inhibition (%)
Negative Control	Carbopol 940^®^ gel	--	0.0 ± 0.0
Positive Control ^a^	Diclofenac in gel	1.0	133.5 ± 12.6 ***
Free Extract ^b^	Extract in gel	1.0	41.2 ± 15.3
Extract-loaded PLGA NPs	Extract-loaded PLGA NPs in gel	1.0	60.7 ± 9.0 *
Extract-loaded PCL NPs	Extract-loaded PCL NPs in gel	1.0	21.7 ± 12.7

**Table 4:** Status of the different parameters evaluated in the plasma of the rats for each group (mean ± S.D.).

Group	TNF-α (pg/mL)	IL-6 (pg/mL)	IL-10 (pg/mL)
Negative Control	168.5 ± 42.2	109.9 ± 1.2	11.4 ± 1.6
Positive Control	95.0 ± 9.6	95.3 ± 9.0	28.8 ± 6.7
Free Extract	133.3 ± 26.5	106.9 ± 7.7	10.7 ± 1.2
Extract-loaded PLGA NPs	117.6 ± 15.1	96.3 ± 7.6	27.3 ± 10.9
Extract-loaded PCL NPs	123.4 ± 17.3	114.6 ± 16.1	18.8 ± 2.9
